# A report on five cases of cholesterol granulomas in the thymus

**DOI:** 10.1186/s44215-024-00159-1

**Published:** 2024-08-21

**Authors:** Ryosuke Matsuda, Naoko Ose, Hideki Nagata, Eiichi Morii, Yasushi Shintani

**Affiliations:** 1 Department of General Thoracic Surgery, Osaka University Graduate School of Medicine, 2-2, Yamadaoka, Suita, Osaka 565-0871 Japan; 2 Department of Pathology, Osaka University Graduate School of Medicine, 2-2, Yamadaoka, Suita, Osaka 565-0871 Japan

**Keywords:** Cholesterol granuloma (CG), Mediastinal tumor, Thymus

## Abstract

**Background:**

Cholesterol granuloma (CG) is a benign entity characterized by the presence of cholesterol crystals and foreign body giant cells. This condition can be attributed to cholesterol crystals that are deposited in the tissues and react with foreign body giant cells, resulting in granuloma formation. Lesions commonly develop in the otolaryngeal region, such as the middle ear. However, crystals rarely form in the thymus, accounting for 1% of all mediastinal tumors. Herein, we present five cases of mediastinal CG.

**Case presentation:**

The patients were aged 49–61 (mean, 55.4) years. Among them, three were men and two women. One patient had fever, and four patients were asymptomatic. The patients’ lesions were detected during follow-up of other diseases or medical examinations showing the presence of abnormal chest shadows. The patients did not have a history of trauma or surgery. All lesions were located within the thymus gland. Three patients presented with multifocal lesions and two with a single lesion. Four patients had contrast effect on computed tomography scan. Four patients had abnormal fluorodeoxyglucose accumulation (mean maximum standardized uptake value, 4.67) on positron emission tomography-computed tomography. Four patients underwent complete surgical resection. The size of the resected lesions ranged from 1.8 to 5.1 (mean, 3.24) cm. Histologically, all patients presented with small nodules with cholesterol clefts and foreign body giant cells and histiocyte infiltration within the thymic tissue. The postoperative course was excellent. None of the patients who underwent complete resection presented with recurrence. Moreover, the patient who underwent partial resection did not have lesion enlargement.

**Conclusions:**

CG in the thymus is clinically challenging to differentiate from malignant lesions, and histologic diagnosis via surgical resection is required.

## Background

Cholesterol granuloma (CG) is a benign entity characterized by the presence of cholesterol crystals and foreign body giant cells [1]. This condition can be attributed to cholesterol crystals that are deposited in the tissues and react with foreign body giant cells, resulting in granuloma formation [1–6]. Lesions commonly develop in the otolaryngological field, such as the middle ear. However, crystals rarely form within the thymus [7]. The incidence rate of mediastinal tumors is 1% [2]. Herein, we present five cases of cholesterol granuloma in the thymus at our institution within the last 4 years.

## Case presentation

**Fig. 1:**
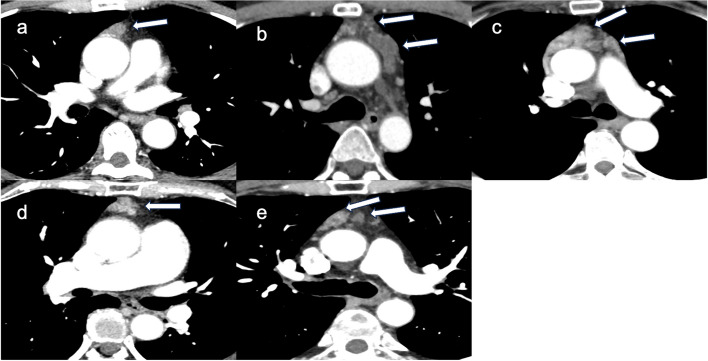
Contrast-enhanced CT scan findings showed the following: Case 1, A 1.2 × 1.2 × 1.7-cm nodule with contrast effect was observed in the anterior mediastinum (**a**); Case 2, Two cystic lesions of irregular morphology were observed in the anterior mediastinum. They were multifocal without contrast effect (**b**); Case 3, A small nodular mass with contrast enhancement was observed in the anterior mediastinum (**c**); Case 4, The size of the irregular mass was 3.0 × 1.2 × 3.5 cm with contrast effect (**d**); Case 5, Multiple cystic masses and nodules with a diameter of up to approximately 2 cm were found in the anterior mediastinum. The interior did not present with contrast effect, and the tumor margins had contrast effects in the form of rings (**e**)

**Fig. 2:**
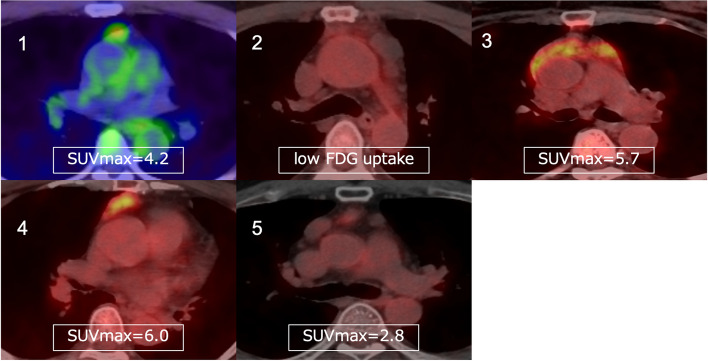
PET-CT scan findings Faint accumulation was only observed in case 2. Meanwhile, evident accumulation was noted in the other cases

**Fig. 3:**
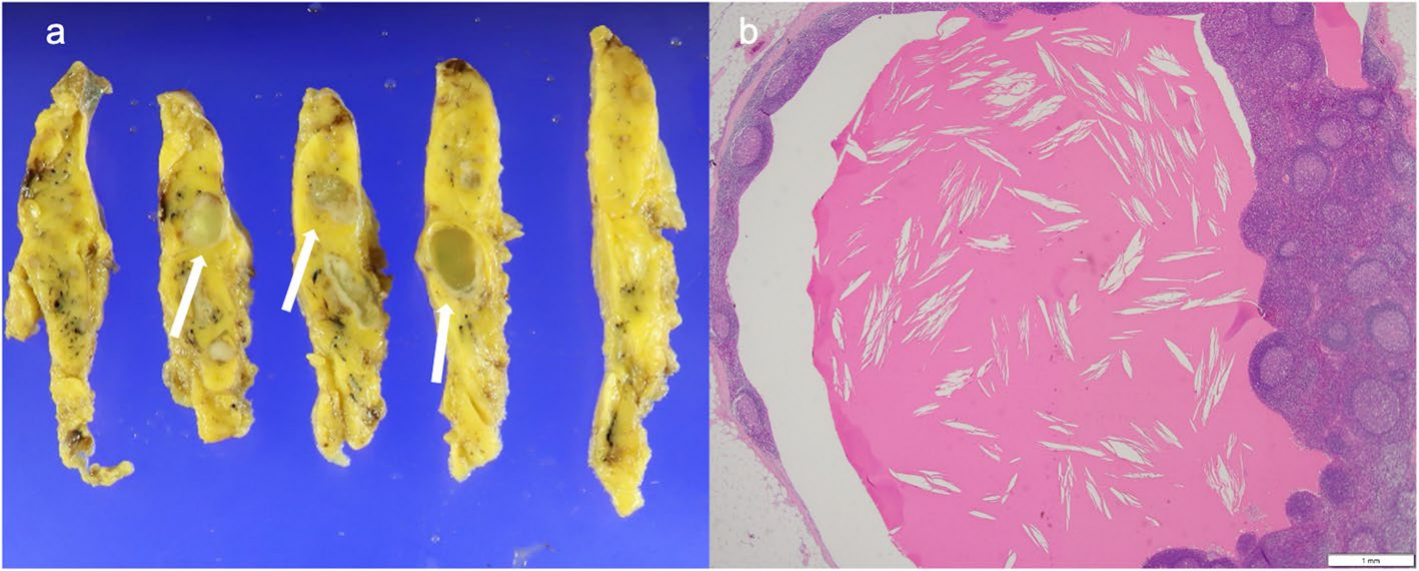
Pathological findings (case 5) showed the following: Macroscopic findings, Multiple substantial cystic lesions were observed in the thymus. The size of the largest lesion was 1.7 × 1.2 × 1.8 cm (arrow) (**a**); Histological findings, A cystic lesion containing cholesterol crystals and multinucleated giant cells was observed in the thymic tissue with hyperplasia of lymph follicles against a background of adipose tissue.(Hematoxylin and Eosin staining, × 20) (**b**)

In total, 5 of 289 patients with mediastinal tumors who underwent surgery at the department from 2020 to 2023 were diagnosed with thymic CG. Table 1 shows the summary of mediastinal CG cases. The patients were aged 49–61 (mean, 55.4) years. Among them, three were men and two women. None of the patients had a history of trauma or surgery. Four patients were asymptomatic, and one had symptoms such as fever. The patients’ inflammatory marker; tumor marker (such as carcinoembryonic antigen, progastrin-releasing peptide, and cytokeratin fragment); soluble interleukin-2 receptor; and antiacetylcholine receptor antibody levels were within normal ranges. All lesions were in the anterior mediastinum. In case 2, the tumor lesion had not changed, though it had gradually grown over time in cases 4 and 5. In cases 1 and 3, it had not been followed preoperatively. Three patients presented with multiple lesions and two with a single lesion. Four patients had contrast effects on contrast-enhanced computed tomography (CT) scan (Fig. 1) and abnormal fluorodeoxyglucose (FDG) accumulation (mean maximum standardized uptake value (SUVmax, 4.7 ± 1.3) on positron emission tomography (PET)-CT (Fig. 2). Preoperative radiological diagnoses varied, including thymoma, thymic carcinoid, thymic carcinoma, multilocular thymic cyst, lymphofollicular thymic hyperplasia, follicular lymphoma, malignant lymphoma, and mucosa-associated lymphoid tissue lymphoma. One patient in case 3 underwent partial resection of multiple lesions for biopsy due to suspected malignant lymphoma based on 3 weeks of persistent fever and the abnormal accumulation on PET-CT. The intraoperative diagnosis was reactive lymphoid hyperplasia. The fever was finally diagnosed as being caused by rheumatoid arthritis. Other patients underwent total thymectomy. Four of the lesions resected were substantial nodules, and one was a cystic lesion. Histopathological examination revealed small nodules with cholesterol clefts, large foreign body cells, and histiocytic infiltrates within the thymic tissue. Hence, a diagnosis of CG was made. Some lesions were accompanied by blood and foci of hemosiderin-phagocytosed histiocytes (Fig. 3). The postoperative course was excellent. None of the four patients who underwent complete resection presented with recurrence. One case that was terminated by biopsy showed no evident lesion enlargement on imaging.

**Table 1:**
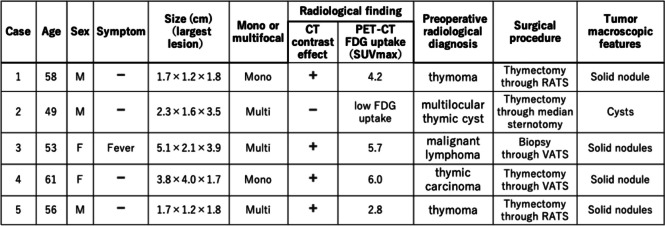
Summary of mediastinal cholesterol granuloma cases at our institution

## Discussion and conclusions

CG are benign masses containing cholesterol crystals and multinucleated giant cells [1]. These lesions were first described by Manasse in 1894 [8]. They are commonly found in the middle ear or paranasal sinuses but rarely in the mediastinum. The overall incidence rate of mediastinal tumors is approximately 1% [2]. In our cases, the incidence was 1.7% in 5 cases out of 289 total mediastinal tumor surgeries. When limited to 126 thymic epithelial tumors, the incidence was 4%. Only 15 cases of CG in the anterior mediastinum were reported from 1973 to 2023 [1–7, 9–13]. Table 2 shows the previously reported cases of CG in the mediastinum. These lesions are more common in men, with a predilection for patients in their 50 s–60 s, and are often detected incidentally during health checkups or other medical examinations in patients without specific symptoms [2]. Similar results were found in our cases.

**Table 2:**
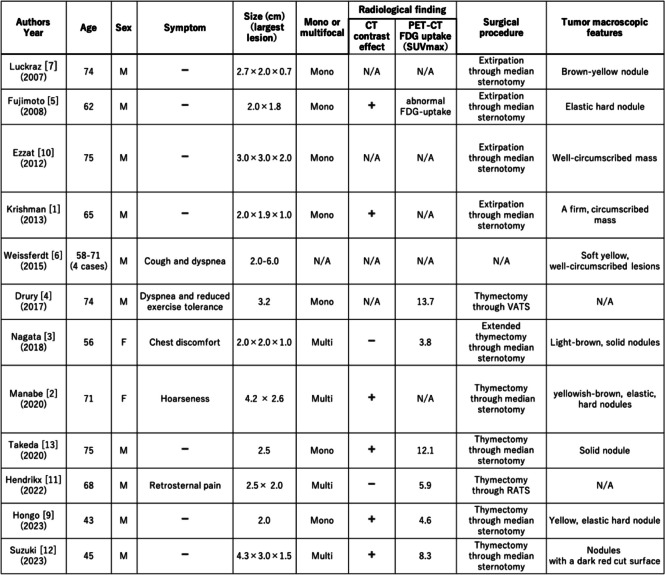
Previously reported cases of cholesterol granuloma in the mediastinum

The exact pathogenesis of the disease is still unknown. However, it involves local microhemorrhage due to vascular injury caused by trauma, chronic inflammation, or dyslipidemia, which induces large foreign body cell reaction and deposition of cholesterol clefts that stimulate granuloma formation [1–6]. In previous reports, a history of trauma, dyslipidemia and diabetes mellitus, which are likely to cause microvascular damage, and cardiovascular disorders have been identified in several cases [2]. However, its pathogenesis [2] and etiology are not clear. In the current study, in case 1, the patient had hypertension. In case 2, the patient had hypertension, dyslipidemia, and cardiovascular disease. However, in other cases, the patients did not present with comorbidities.

Although most cases involved masses on chest CT scan, scattered nodules or mottled calcifications can be observed in some patients [5, 10]. In previous reports, some lesions developed in the posterior mediastinum. However, most of them formed in the anterior mediastinum [2]. The patients commonly presented with single lesions, and only seven cases including our three cases involved multiple lesions [2, 3, 11, 12]. PET-CT often shows a significant FDG uptake caused by granulomatous inflammation [5]. An abnormal FDG accumulation (mean SUVmax, 4.67) was observed in the lesions in our case. On T1- and T2-enhanced magnetic resonance imaging, the lesions had an equal to low signal compared with the muscle [5]. These imaging features are similar to those of thymic epithelial tumors, thereby making preoperative diagnosis challenging. In our cases, the preoperative differential diagnoses based on the imaging findings were diseases such as thymoma, thymic carcinoid, thymic carcinoma, multilocular thymic cyst, lymphofollicular thymic hyperplasia, follicular lymphoma, malignant lymphoma, and mucosa-associated lymphoid tissue lymphoma. However, CG could not be predicted preoperatively. There are no reports on CG of the thymus diagnosed preoperatively, and diagnostic imaging is challenging. By contrast, the histopathology of the lesions is characterized by the presence of several cholesterol crystals surrounded by foreign body giant cells. Further, the lesions can be easily differentiated from a thymic epithelial tumor.

In terms of treatment, complete resection is performed, and recurrence has not been reported [5, 10]. There is no report on resection of only a portion of multiple lesions, and complete resection is preferred. However, in case 3 of the current study, the lesion size did not increase during follow-up, and we planned to cautiously monitor the long-term prognosis in this case. In some cases, the lesions adhered to adjacent organs, partly due to inflammation [11]. Therefore, intraoperative manipulation should be performed with caution.

CG is challenging to differentiate from thymoma and other malignant conditions because of its predilection for lesions in the anterior mediastinum and a high FDG uptake on PET-CT. Recurrence after complete resection has not been reported, and surgical resection is required for both the diagnosis and treatment of the disease.

## Data Availability

Not applicable.

## Abbreviations

CG: Cholesterol granulomas
CT: Computed tomography
FDG: Fluorodeoxyglucose
PET: Positron emission tomography
SUVmax: Maximum standardized uptake value
